# Dietary total antioxidant capacity significantly interacts with 6-P21 rs2010963 gene polymorphisms in terms of cardio-metabolic risk factors in patients with metabolic syndrome

**DOI:** 10.1186/s13104-020-04993-8

**Published:** 2020-03-11

**Authors:** Mahdieh Abbasalizad Farhangi

**Affiliations:** 1 Nutrition Research Center, Tabriz University of Medical Sciences, Tabriz, Iran; 2 Drug Applied Research Center, Tabriz University of Medical Sciences, Tabriz, Iran

**Keywords:** Dietary antioxidant capacity, Metabolic syndrome, 6P21 rs2010963 gene polymorphisms

## Abstract

**Objective:**

Gene- nutrient interaction might possibly be involved in the pathogenesis of metabolic syndrome and its components. In the current report, the association between antioxidant potential of the diet with 6P21 rs2010963 gene polymorphism in patients with metabolic syndrome has been evaluated. Two hundred fifty-four patients with metabolic syndrome were enrolled. Total dietary antioxidant capacity (TAC) has been estimated and anthropometric assessments were assessed. Biochemical assays including serum glucose, matrix metalloproteinase-3, liver enzymes and lipid profiles were also assessed. Polymerase chain reaction- restriction fragment length polymorphism (PCR–RFLP) method was used for determination of 6P21 rs2010963 polymorphism.

**Results:**

Dietary vitamin E score was significantly higher in GC genotype compared with other genotypes (P = 0.035). Patients in CC genotype of 6P21 rs2010963 had significantly higher body mass index (BMI), fasting blood sugar and liver enzymes (P < 0.05). Being in the higher dietary TAC scores was also associated with lower liver enzymes. The interaction between 6P21 rs2010963 and dietary TAC significantly affected BMI, FBS and diastolic blood pressure (P < 0.05). According to our findings the CC genotype of 6P21 rs2010963 could be considered as the possible risk factor for obesity and metabolic disorders among patients with metabolic syndrome.

## Introduction

Metabolic syndrome, known as “insulin resistance syndrome”, “American syndrome”, and “syndrome X” is associated with numerous abnormalities including type 2 diabetes, cardiovascular disease, chronic kidney disease and mental disorders [1–3].

The prevalence of metabolic syndrome is increasing worldwide, and it has been estimated that for the adult population to be about 20 to 25% [4, 5]. In Iran also the prevalence of disease is high and according to the data from two rounds of the Surveillance of Risk Factors of Non-communicable Diseases national surveys conducted in 2007 and 2011, approximately one-third of Iranian adult population had metabolic syndrome (~ 32–36%) [6].

Metabolic syndrome is often accompanied by an imbalance between the production and inactivation of reactive oxygen species leading to oxidative stress. Reactive oxygen species can act as double-edged swords; while they play an essential role in multiple physiological systems, under conditions of oxidative stress, they contribute to cellular dysfunction [7, 8].

Diet and dietary habits play a crucial role in the pathogenesis of metabolic syndrome and dietary habits are potential determinants of the disease severity and several studies have highlighted the protective role of dietary antioxidants against metabolic syndrome [9–11]. Recently, the role of dietary total antioxidant capacity (TAC) as a whole indicator of antioxidant potential of diet has been attracted much attention. Puchau et al. [12], reported TAC is a novel indicator of the overall dietray antioxidant potential and is a useful tool to assess diet-disease relationship. Other study by Bahadoran et al. [13] demonstrated that higher TAC of diet is associated with reduced occurrence of metabolic syndrome ingredients in healthy individuals studied in Tehran Lipid and Glucose Study.

Recently, the gene-nutrient interactions and their role if pathogenesis of disease has attracted much attention. It has been suggested that gene variants of single nucleotide (SNPs) polymorphisms in the chromosome 6p21.3 rs2010963 is associated with insulin resistance [14–17] and its targeting, inhibition and blockage is a novel treatment for insulin resistance in type 2 diabetes and metabolic syndrome [18]. However, no study is available in evaluation of the association between 6P21 rs2010963 polymorphism and TAC with metabolic syndrome components and inflammatory biomarkers in patients with metabolic syndrome. Therefore the primary objectives of the current study was to evaluate the interaction between dietary total antioxidant capacity and rs2010963 polymorphism to affect components of metabolic syndrome, adiponectin and matrix metallo-proteinase (MMP)-3 in patients with metabolic syndrome.

## Main text

### Methods

#### Design and participants

A total number of 254 patients with metabolic syndrome who were referred to out-patients clinics of Tabriz University of Medical Sciences were involved in the current study. National Cholesterol Education Program’s Adult Treatment Panel III (NCEP-ATP III) criteria was used for definition of metabolic syndrome [19]. The subjects were aged 25 years old and above and were living in Tabriz. We excluded patients with any history of cardiovascular disorders, renal diseases, type 2 diabetes mellitus and cancer. Also, pregnant women and patients who received anti-hypertensive or anti-hyperlipidemia medications, antioxidant supplements, and patients who were at special diets (e.g. caloric restriction) at least 3 months prior participation in the study. The patients’ recruitment and the study duration was from September 2014 to December 2018. The study protocol has been approved by the ethics committee of Tabriz University of Medical Sciences (IR.TBZMED.REC.1398.460). Anthropometric assessments were performed as explained previously [20, 21].

#### Biochemical measurements

Enzymatic colorimetric methods using routine biochemical kits (Pars–Azmoon, Tehran–Iran) were used for assessment of serum aspartate aminotransferase (AST), alanine aminotransferase (ALT), total cholesterol (TC), fasting blood sugar (FBS), triglyceride (TG), high density lipoprotein cholesterol (HDL-C) and low density lipoprotein cholesterol (LDL-C). Serum insulin was also analyzed with enzyme linked immunosorbent assay method (ELISA- Monobind Insulin AccuBind, CA 92630, USA) with the sensitivity of 0.75 µIU/ml and mean inter and intra assay coefficients of variation (CV) as < 9.8% and < 8% respectively. Serum MMP-3 was also analyzed by ELISA method (Hangzhou Eastbiopharm Co, USA) with the sensitivity and assay range of 0.01 and 0.05–10 ng/ml respectively. Serum adiponectin was also analyzed by ELISA method (AviBion, Fin-01720 Vantaa, Finland). Insulin resistance was assessed by the homeostasis model assessment of insulin resistance (HOMA-IR) as follows: HOMA-IR: [glucose (mmol/l) × insulin (mU/l)]/405. High HOMA-IR scores denote high insulin resistance. The quantitative insulin check index (QUICKI) was calculated as: 1/(log fasting insulin (U/l) + log fasting glucose (mg/dl)) while higher QUICKI values indicate greater insulin sensitivity [22, 23].

#### Measurement of total dietary antioxidant capacity

A validated semi-quantitative 147 item food-frequency questionnaire (FFQ) was used for dietary assessment [24]. Total dietary antioxidant capacity (TAC) was calculated according to a method first described by Rivas et al. [25, 26]. The intake of several important antioxidants including vitamin A, vitamin E and vitamin C, zinc and selenium were assessed separately by assigning a score of 0 or 1. This scoring was based on the comparison of nutrient intake with the dairy recommended intake of nutrients (RDA). When the intake was below 2/3 of the RDA, 0 score was signed. While in the dietary intake of nutrient higher than 2/3 RDA of that nutrient, the assigned score was 1. Therefore, the total dietary antioxidant intake (TAC) was ranged between 0 (very poor) to 5 (high quality).

#### Determination of the 6P21.3 rs2010963 genotypes

Polymerase chain reaction- restriction fragment length polymorphism (PCR-RFLP) was used to determine the 6 P21.3 rs2010963 polymorphism status with technical specifications. Briefly, after amplification of genomic DNA with denaturation, annealing and extension, the primer pairs and fluorogenic probes with the sequence of: Forward: 5′-TTGCTTGCCATTCCC-CACTTGA-3 and Backward: 5′-CCGAAGCGAGAACAGCCCAGAA-3′ were used for determination of VEGF polymorphisms. A total volume of 10 μL including 100 ng DNA, 25 μL Taq PCR master mix RED (Ampliqon; Denmark), 2.5 μL primers and 12.5 μL deionized water was the final volume of PCR reaction under the following conditions: denaturation at 95 °C for 5 min, 35 cycles at 94 °C for 1 min, 60 °C for 1.5 min (annealing) and 72 °C for 2 min (extension). The final extension was done at 72 °C for 10 min. The digestion was performed by Bsm FI restriction nuclease (New England Biolabs, USA). All products were separated by electrophoresis using 1% agarose gel stained with ethidium bromide. For the 6P21.3 rs2010963 genotypes the uncut fragment was 469 bp (C allele) and digestion products were 195 bp and 274 bp (G allele).

#### Statistical analyses

Statistical package for social analysis (version 18, SPSS Inc., Chicago, IL, USA) was used for data analysis. Kolmogorov–Smirnov test was used for normality check of data. Chi- square test, independent sample *t*-test and analysis of variance (ANOVA) were used for the comparison of continuous and discrete variables. Analysis of covariance (ANCOVA) was also used for adjustment of the confounders (e.g. age and gender). Data are expressed as mean ± SD. A two-sided *P* value less than 0.05 was considered significant. The gene-nutrient interaction was also obtained by general linear model (GLM) with adjustment for possible confounders.

## Results

Additional file 1: Table S1 presents daily intakes of antioxidants in study population. As shown, majority of subjects received adequate amounts of zinc, selenium and vitamin C as low percent of them received lower than 2/3 RDA. However, approximately more than 50% (~ 55, 56%) of individuals received poor amounts of vitamin E and vitamin A in their daily dietary intakes. In Table 1 metabolic profile and dietary antioxidants score in different 6P21 rs2010963 genotypes are presented. As shown, higher BMI, FBS, AST and ALT concentrations were observed in different 6P21 rs2010963 genotypes (P < 0.05). Moreover, patients in GC genotype had the highest dietary vitamin E score compared with other genotypes (P = 0.035). No statistically significant difference was observed between other biochemical parameters and antioxidant scores among different 6P21 rs2010963 polymorphism genotypes. In comparison of the metabolic and biochemical parameters between different scores of TAC among participants (Table 2), gender was a significant predictor of TAC (P = 0.031); while higher percentage of male participants were in lower than median TAC scores compared with females (P = 0.031). Accordingly, dietary zinc, vitamin A and vitamin C antioxidant scores and total dietary antioxidant score among females were significantly higher than males (P < 0.05 and P < 0.001); whereas, no significant difference between selenium and vitamin E score was identified (Additional file 1: Figure S1).

**Table 1:** Metabolic profile and dietary antioxidants score among study population according to 6P21 rs2010963 genotypes

	6P21 rs2010963 genotypes			P value
	GG (N = 72)	GC (N = 132)	CC (N = 46)	
Age (y)	42.06 ± 9.71	41.24 ± 10.17	43.73 ± 8.65	0.48
Male (%)	54.5	55.1	52.6	0.91
BMI (kg/m^2^)	30.74 ± 5.64	30.37 ± 5.37	31.75 ± 5.79	*0.027*
WHR	0.93 ± 0.07	0.93 ± 0.07	0.93 ± 0.06	0.96
SBP (mmHg)	128.40 ± 11.79	132.70 ± 10.98	132.08 ± 11.95	0.33
DBP (mmHg)	86.81 ± 5.67	88.68 ± 6.04	89.58 ± 7.21	0.37
FBS (mg/dl)	89.03 ± 9.47	88.05 ± 10.73	91.73 ± 19.35	*0.047*
TC (mg/dl)	190.57 ± 39.19	193.82 ± 40.83	188.73 ± 30.84	0.85
TG (mg/dl)	187.24 ± 74.12	163.00 ± 89.53	182.42 ± 73.04	0.405
LDL (mg/dl)	121.87 ± 31.90	127.27 ± 32.15	119.58 ± 29.83	0.54
HDL (mg/dl)	43.24 ± 12.75	44.85 ± 10.66	45.42 ± 10.18	0.74
AST (IU/l)	19.80 ± 7.38	23.00 ± 7.22	25.51 ± 9.74	*0.037*
ALT (IU/l)	19.66 ± 10.79	24.00 ± 11.36	26.87 ± 12.01	*0.049*
MMP-3 (ng/ml)	3.4 ± 0.98	4.1 ± 0.76	4.9 ± 0.56	0.59
Insulin (µIU/l)	15.47 ± 10.12	17.73 ± 9.87	13.53 ± 3.12	0.87
Adiponectin (ng/ml)	12.04 ± 4.65	14.58 ± 4.93	13.37 ± 3.60	0.24
HOMA-IR	3.44 ± 0.12	3.83 ± 0.28	2.78 ± 1.09	0.88
QUICKI	0.34 ± 0.05	0.33 ± 0.04	0.33 ± 0.01	0.59
Zn score	0.99 ± 0.01	0.95 ± 0.21	0.97 ± 0.02	0.32
Se score	0.97.00 ± 0.01	0.98 ± 0.01	0.96 ± 0.02	0.28
Vitamin A score	0.36 ± 0.14	0.49 ± 0.15	0.29 ± 0.08	0.16
Vitamin C score	0.94 ± 0.22	0.95 ± 0.21	0.96 ± 0.17	0.93
Vitamin E score	0.26 ± 0.11	0.53 ± 0.21	0.32 ± 0.08	*0.035*
Total TAC score	3.51 ± 0.76	3.92 ± 0.92	3.57 ± 0.76	0.12

Italic signifies P < 0.05

*BMI* body mass index, *WC* waist circumference, *WHR* waist to hip ratio *FBS* fasting blood sugar, *TC* total cholesterol, *TG* triglyceride, *HDL* high density lipoprotein cholesterol, *LDL* low density lipoprotein cholesterol, *ALT* alanine aminotransferase, *AST* aspartate aminotransferase, *SBP* systolic blood pressure, *DBP* diastolic blood pressure, *MMP*-3 matrix metalloproteinase-3, *HOMA-IR* homeostatic model assessment of insulin resistance, *QUICKI* Quantitative Insulin Sensitivity Check Index

P value based on independent T-test using equal variable. Continuous variables data are presented based on mean (SD) (the P values for biochemical variables were obtained by ANCOVA adjusted for BMI, age and gender)

**Table 2:** The comparison of metabolic profile among participants according to median TAC score

Variable	TAC score	N	Mean	SD	P value
Age (year)	< median	127	44.21	9.40	*0.04*
	≥ median	127	41.43	10.48	
Male (%)	< median	127	70		*0.031*
	≥ median	127	65.3		
BMI (kg/m^2^)	< median	127	29.71	4.184	0.32
	≥ median	127	30.46	4.96	
FBS (mg/dl)	< median	127	88.41	12.87	0.12
	≥ median	127	91.36	13.16	
TC (mg/dl)	< median	127	192.61	36.20	0.53
	≥ median	127	196.14	39.40	
TG (mg/dl)	< median	127	180.58	101.87	0.82
	≥median	127	176.88	108.80	
HDL (mg/dl)	< median	127	43.22	10.96	0.76
	≥ median	127	42.76	9.93	
LDL (mg/dl)	< median	127	122.34	28.41	0.16
	≥ median	127	128.69	33.66	
ALT (IU/l)	< median	127	29.03	8.63	*0.049*
	≥ median	127	25.07	8.53	
AST (IU/l)	< median	127	32.21	10.48	*0.021*
	≥ median	127	27.44	10.29	
BMI (kg/m^2^)	< median	127	29.71	4.184	0.32
	≥ median	127	30.46	4.96	
SBP (mmHg)	< median	127	130.84	10.21	0.34
	≥ median	127	132.47	10.71	
DBP (mmHg)	< median	127	88.36	5.73	0.94
	≥ median	127	88.29	7.13	
MMP3 (ng/ml)	< median	127	4.46	3.42	0.96
	≥ median	127	4.42	3.45	
Insulin (µIU/l)	< median	127	18.34	27.12	0.83
	≥ median	127	17.05	19.52	
Adiponectin	< median	127	13.81	4.16	0.76
	≥ median	127	14.11	4.95	
HOMR-IR	< median	127	4.00	5.81	0.88
	≥ median	127	3.79	4.50	
QUICKI	< median	127	0.33	0.050	0.77
	≥ median	127	0.34	0.053	

Italic signifies P < 0.05

*BMI* body mass index, *WC* waist circumference, *WHR* waist to hip ratio, *FBS* fasting blood sugar, *TC* total cholesterol *TG* triglyceride, *HDL* high density lipoprotein cholesterol, *LDL* low density lipoprotein cholesterol, *ALT* alanine aminotransferase, *AST* aspartate aminotransferase, *SBP* systolic blood pressure, *DBP* diastolic blood pressure, *MMP*-3 matrix metalloproteinase-3, *HOMA-IR* homeostatic model assessment of insulin resistance, *QUICKI* Quantitative Insulin Sensitivity Check Index

P value based on independent T-test using equal variable. Continuous variables data are presented based on mean (SD) (the P values for biochemical variables were obtained by ANCOVA adjusted for BMI, age and gender)

**Fig. 1:**
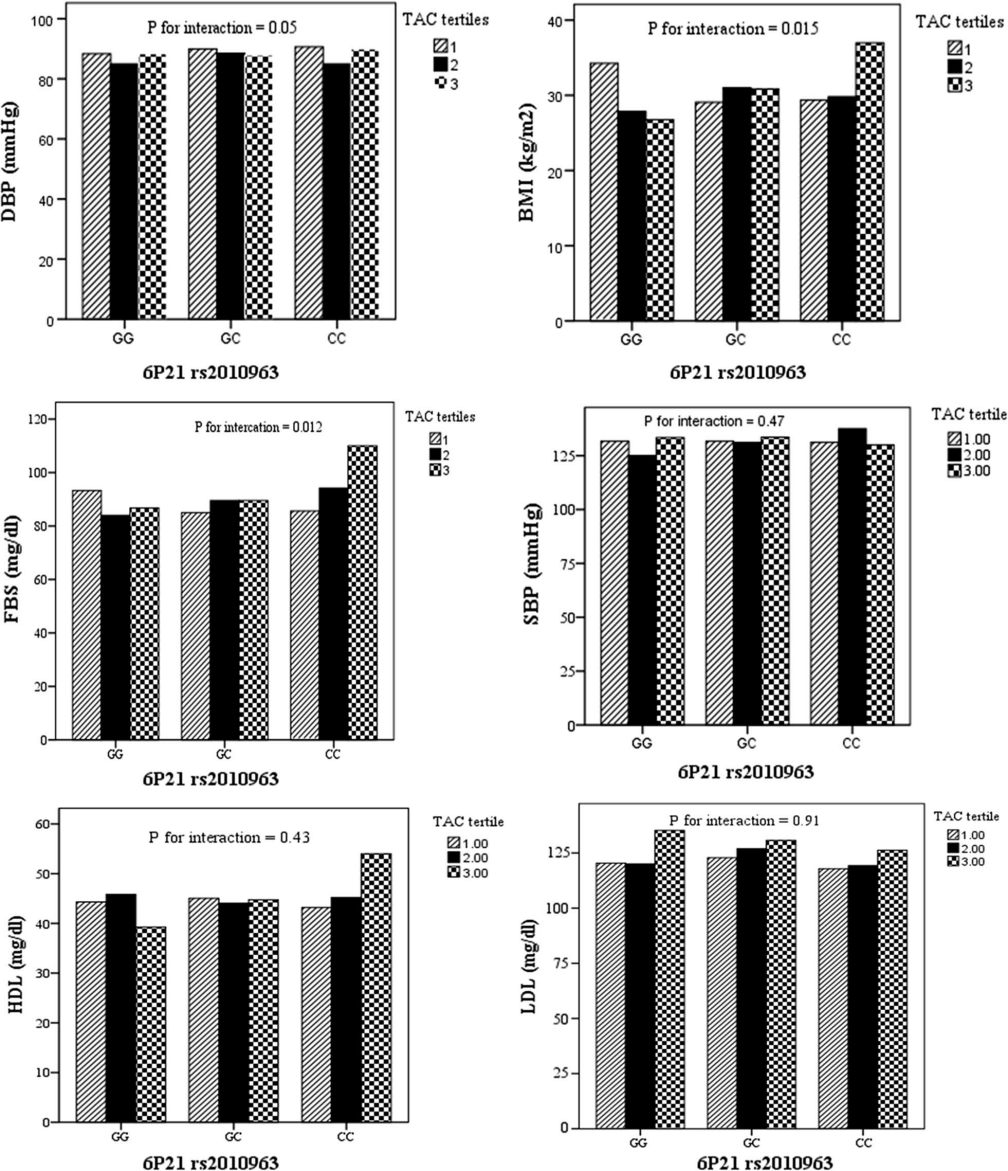

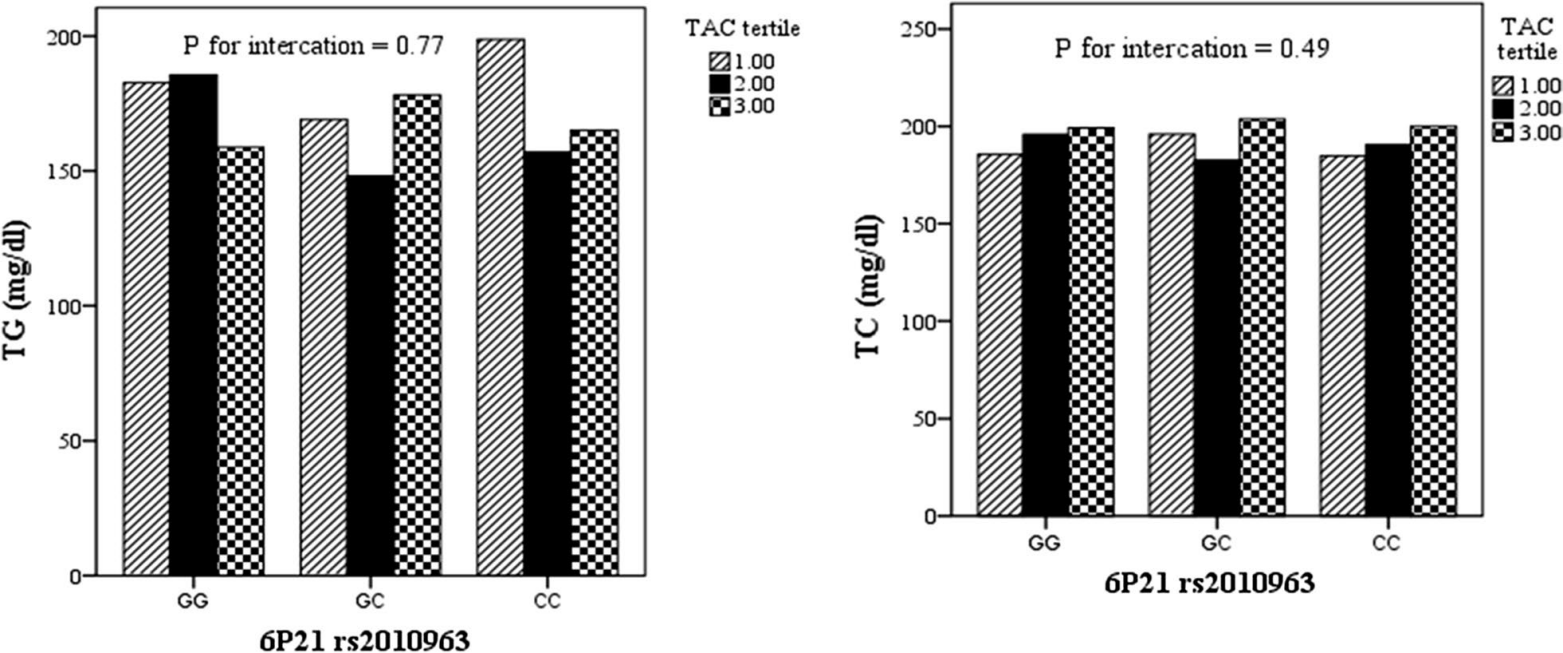
The interaction between chromosome 6 P21 rs2010963 and TAC tertiles (P-for interaction was achieved by general linear model for BMI the covariates were age, gender, and for serum lipids and blood pressure additionally adjusted for BMI. Statistically significant interactions for BMI, FBS and DBP

Moreover, age and liver enzymes were higher in lower than median scores of TAC compared with higher scores (P = 0.04, 0.049 and 0.021 respectively). Figure 1 presents the effects of interaction between dietary antioxidant intake and 6P21 rs2010963 genotypes on anthropometric and biochemical variables. The results of general linear model presented that gene-TAC interaction significantly affects BMI, FBS and DBP in participants (P for interaction = 0.015, 0.05 and 0.012 respectively).

## Discussion

This is the first study, evaluated the possible interaction between dietary antioxidant intake with 6P21 rs2010963 polymorphisms in patients with metabolic syndrome and according to our findings, significantly higher values of BMI, FBS and liver enzymes in patients of CC genotypes of 6P21 rs2010963 polymorphisms and also significant effects of interaction between dietary TAC and 6P21 rs2010963 polymorphisms on BMI, FBS and DBP was identified. Higher TAC scores were accompanied with lower liver enzymes and younger ages. Moreover, female participants had significantly higher antioxidant scores compared with men.

Previous studies also revealed the association between 6P21 rs2010963 genotype with severity and extent of coronary artery disease [27, 28]. In fact, health dietary choices might be inhibitors of 6P21 rs2010963 gene expression [29] while inappropriate dietary habit might induce it [30, 31]. The CC genotype and the C allele of 6P21 rs2010963 gene is considered to be the main risk factor of metabolic disorders, cardiovascular events; Nia SA et al. showed that C allele of rs2010963 polymorphism was related to increase risk for CAD and to 5‐year cardiovascular mortality in Iranian population [32]. In other study also being in CC genotype of 6P21 rs2010963 was associated with chronic heart failure in MERIT-HF study [33]. More interestingly, our findings also presented the interaction of CC genotype with dietary antioxidant intake on BMI, blood sugar and diastolic blood pressure.

According to our results, females had higher total dietary antioxidant scores mainly because of higher dietary intakes of zinc, vitamin A and vitamin C. In the study by Detopoulou et al. [34] women had non-significantly higher dietary ferric reducing antioxidant power and higher Trolox-equivalent antioxidant capacity compared with men. Moreover, in a population based study by Chen R [35] dietary antioxidant intakes of vitamin C and β-carotene were higher in women compared with men. The similar findings was also reported in the study by Bates et al. [36] reporting the higher energy adjusted intake of retinol, vitamin C and calcium in women compared with men and better dietary choices and intakes among younger ages; or other study reported higher vitamin E intake and its consequently better performance on a psychomotor speed test compared with men [37]. Although several discrepancies are also present and several other studies also reported the higher antioxidant intakes among men compared with women [38], the possible reason is better food choices and healthy dietary habits among women possibly because of women’s high motivation to be involved in weight control programs and their higher adherence in healthy eating patterns as previously reported by Wardle et al. [39]. In our study, high dietary antioxidant score was associated with lower AST and ALT as expected and in agreement of previous human or experimental reports [40–42].

## Conclusion

According to our findings, CC genotype of 6P21 rs2010963 was considered as possible risk factor of metabolic disorders. Moreover, 6P21 rs2010963 showed significant interactions in terms of BMI, fasting blood sugar and diastolic blood pressure in patients with metabolic syndrome.

## Limitation

The cross-sectional design of the work might it impossible to elucidate the causality. Also, measurement of other SNPs and their association with 6P21 rs2010963 genotype could be helpful in explaining gene-TAC interactions.

## Supplementary information

**Additional file 1: Table S1.** Dietary antioxidant daily intakes in study population. **Figure S1.** The comparison of dietary antioxidant scores among male and female patients with metabolic syndrome (* denotes the p-values of independent t-test lower than 0.05; ** denotes the p-values of independent t-test lower than 0.001).

## Data Availability

The datasets used and/or analyzed during the current study available from the corresponding author on reasonable request.

## Abbreviations

ANOVA: Analysis of variance
CAD: Coronary artery disease
CVD: Cardiovascular disease
HDL: High density lipoprotein cholesterol
LDL: Low density lipoprotein cholesterol
TC: Total cholesterol
TNF-α: Tumor necrosis factor-α
IL: Interleukin
FSG: Fasting serum glucose
MMP-3: Matrix metalloproteinase-3
PCR–RFLP: Polymerase chain reaction-restriction fragments length polymorphism
TAC: Total antioxidant capacity
TLGS: Tehran Lipid and Glucose Study
VEGF: Vascular endothelial growth factor
NCEP-ATP III: National Cholesterol Education Program’s Adult Treatment Panel III
DBP: Diastolic blood pressure
SBP: Systolic blood pressure
ROS: Reactive oxygen species
