# Supplementary material for: Dietary total antioxidant capacity significantly interacts with 6-P21 rs2010963 gene polymorphisms in terms of cardio-metabolic risk factors in patients with metabolic syndrome
Source: BMC Res Notes. 2020 Mar 11;13:145. doi: 10.1186/s13104-020-04993-8 (PMC7066746; doi:10.1186/s13104-020-04993-8)
Supplement: Supplementary file 1 — Additional file 1: Table S1. Dietary antioxidant daily intakes in study population. Figure S1. The comparison of dietary antioxidant scores among male and female patients with metabolic syndrome (* denotes the p-values of independent t-test lower than 0.05; ** denotes the p-values of independent t-test lower than 0.001). [file 13104_2020_4993_MOESM1_ESM.docx]

**Additional file 1**

**Title: Dietary total antioxidant capacity significantly interacts with 6-P21 rs2010963 gene polymorphisms in terms of cardio-metabolic risk factors in patients with metabolic syndrome**

**Short title:** Dietary antioxidant capacity and 6 P21 rs2010963 gene polymorphisms

Mahdieh Abbasalizad Farhangi^1^, Leila Nikniaz ^2^, Zeinab Nikniaz ^3^

^1^ Drug Applied Research Center, Tabriz University of Medical Sciences, Tabriz, Iran

^2^ Tabriz Health Services Management Research Center, Health Management and Safety Promotion Research Institute, Tabriz University of Medical Sciences, Tabriz-Iran

^3^ Liver and Gastrointestinal Disease Research Center, Tabriz University of Medical Sciences, Tabriz, Iran.

***Corresponding Author**, Email address: abbasalizad_m@yahoo.com, Tel: +98 0413 3357580

**Table S1. Dietary antioxidant daily intakes in study population**

|  | **Minimum** | **Maximum** | **Mean** | **SD** | **% Sample < 2/3 RDA** |
| --- | --- | --- | --- | --- | --- |
| **Zn (mg)** | 4.87 | 81.38 | 15.19 | 8.24 | 8 (3.2) |
| **Se (µg)** | 53.31 | 493.06 | 150.98 | 63.86 | 2 (0.6) |
| **Vitamin A (RAE)** | 84.56 | 3470.18 | 838.19 | 508.63 | 141 (55.7) |
| **Vitamin C (mg)** | 31.27 | 997.66 | 312.22 | 212.83 | 10 (3.8) |
| **Vitamin E (mg)** | 4.06 | 36.65 | 14.62 | 5.60 | 138 (54.4) |

**RAE, retinol acid equivalents. RDA, recommended dietary allowances**

**Figure S1.** The comparison of dietary antioxidant scores among male and female patients with metabolic syndrome (* denotes the p-values of independent t-test lower than 0.05; ** denotes the p-values of independent t-test lower than 0.001).
